# Sickle Cell Disease: Current Understanding and Future Options

**DOI:** 10.3390/jcm12185943

**Published:** 2023-09-13

**Authors:** Christos Varelas, Eleni Gavriilaki

**Affiliations:** 1 Hematology Department—BMT Unit, G .Papanicolaou Hospital, 57010 Thessaloniki, Greece; 2 2nd Propedeutic Department of Internal Medicine, Aristotle University of Thessaloniki, 54644 Thessaloniki, Greece

Sickle cell disease (SCD) is a prevalent inherited hemoglobin disorder encompassing a cluster of congenital hemolytic anemias, each distinguished by the prevalence of sickle hemoglobin (HbS) [1]. Anemia, predisposition to mainly bacterial infections and complications that include vaso-occlusive crisis (VOC) and delayed hemolytic transfusion reaction (DHTR) are its main features, with the two latter being responsible for the increased rates of morbidity and mortality despite the use of hydroxyurea as a standard of care [2,3]. The primary pathophysiological mechanism of the disease involves hemolysis resulting from the interaction of sickle cells with neutrophils, platelets, or endothelial cells in small blood vessels. Studies of small cohorts of SCD patients revealed complement activation, although the exact mechanism remains largely unknown. Therefore, it is important to predict the group of patients that could potentially benefit from complement inhibition. Eculizumab is the only complement inhibitor that has been used with beneficial results in DHTR and VOC to date [4]. 

Numerous novel agents are currently undergoing clinical development or have been introduced into clinical practice. In addition, the attempts to reduce long-term complications and enhance quality of life continue. This Special Issue aims to delineate both our current understanding and future options in SCD. 

All articles submitted to this Special Issue underwent a meticulous peer review process. Ultimately, two articles, two brief reports and one review were published. These five manuscripts are discussed below.

(i) Forte and colleagues provided important data on the timely issue of COVID-19 and SCD, updating outcomes after two years of the pandemic [5].
(ii) Tsitsikas and colleagues presented the rate of dental extractions in SCD, providing up-to-date knowledge on an understudied complication [6].
(iii) Biswas and colleagues reviewed the role of mitochondria as an emerging consequential in SCD, highlighting pathophysiological and therapeutic challenges [7].
(iv) Kuo and colleagues reported that thromboprophylaxis reduced venous thromboembolism in SCD patients with central venous access devices, studying a large retrospective cohort [8].
(v) Gavriilaki and colleagues studied the immune response of adult SCD patients after their vaccination against COVID-19, providing the experience of a Greek center.

Considering the multi-faceted challenges of SCD, we hope that this Special Issue will inspire researchers and clinicians to continue their explorations into novel advances in this field [9].
